# On Riemann-Liouville integrals and Caputo Fractional derivatives via strongly modified (*p, h*)-convex functions

**DOI:** 10.1371/journal.pone.0311386

**Published:** 2024-10-15

**Authors:** Ammara Nosheen, Khuram Ali Khan, Mudassir Hussain Bukhari, Michael Kikomba Kahungu, A. F. Aljohani

**Affiliations:** 1 Department of Mathematics, University of Sargodha, Sargodha, Pakistan; 2 Higher Institute of Education of Gombe (ISP Gombe), Kinshasa, Congo; 3 Department of Mathematics, Faculty of Science, University of Tabuk, Tabuk, Saudi Arabia; Universiti Tun Hussein Onn Malaysia, MALAYSIA

## Abstract

The paper introduces a new class of convexity named strongly modified (*p*, *h*)-convex functions and establishes various properties of these functions, providing a comprehensive understanding of their behavior and characteristics. Additionally, the paper investigates Schur inequality and Hermite-Hadamard (H-H) inequalities for this new class of convexity. Also, H-H inequalities are proved within context of Riemann-Liouville integrals and Caputo Fractional derivatives. The efficiency and feasibility of Schur inequality and H-H inequalities are supported by incorporating multiple illustrations, that demonstrate the applicability of strongly modified (*p*, *h*)-convex functions. The results contribute to the field of mathematical analysis and provide valuable insights into the properties and applications of strongly modified (*p*, *h*)-convex functions.

## 1 Introduction

The concept of fractional calculus was pioneered by Niels Henrik Abel, and the foundation of fractional calculus as an independent subject was laid by Liouville [1]. Fractional calculus is pivotal in applied mathematics and mathematical analysis [2]. It makes it possible to find the solution of fractional derivatives and fractional integrals of any order.

In mathematical analysis, convex analysis plays a crucial role in exploring the properties and applications of convex functions [3, 4]. Convex functions are pivotal in many fields such as engineering [5], economics [6], geometry [7] and mathematical optimization [8]. The applications of convex functions to the special functions are presented in [9].

In the field of mathematics, inequalities are very crucial. In convex analysis, Hermite-Hadamard (H-H) inequalities, which were established by Charles Hermite and Jacques Hadamard in 1885 [5], are of utmost importance.

For a convex function $\xi :B_{1}\subseteq R\to R$, the H-H inequality [10] is given as,

$$\begin{array}{c} \xi (\frac{u_{1}+u_{2}}{2})\leq \frac{1}{u_{2}-u_{1}}\int_{u_{1}}^{u_{2}}\xi \left(k\right)\;dk\leq \frac{\xi \left(u_{1}\right)+\xi \left(u_{2}\right)}{2},\;\;where\;u_{1},u_{2}\in B_{1}. \end{array}$$

H-H inequalities can establish maximum and minimum values of functions over an interval. This behaviour makes them applicable in multiple fields. For example, in the field of probability theory, H-H inequalities establish bounds for the occurrence of a particular event [11]. Also, in image processing, H-H inequalities are utilized to ensure image quality by fixing pixels within particular limits [12]. Also, the H-H type inequalities for subadditive functions is provided in [13].

Motivated by the applications of H-H inequalities in multiple disciplines and the work done by Angulo, H., Giménez, J., Moros, A. M., & Nikodem, K on strongly *h*-convex functions in [14], Feng, B., et al on modified (*p*, *h*)-convex functions in [15], Zhang et al. on *p*-convex functions in [16, 17], and Toader G., on the family of modified *h*-convex function in [18], we have introduced a novel class of convex functions which generalizes these motivated classes and have provided its applications in the form of Schur inequality and H-H inequalities.

To achieve the goals, the paper is structured in the following order: Some important definitions are reviewed in Section 2. The notion of strongly modified (*p*, *h*)-convex function is introduced in Section 3. Also, some basic properties are proved for this novel class of convexity. The Schur inequality is presented in Section 4 for this newly defined class of convex functions. Section 5 demonstrates the proofs of the H-H inequalities for strongly modified (*p*, *h*)-convex function. Lastly, Section 6 provides a comprehensive summary of the entire research work.

## 2 Preliminaries

The following are some definitions which are useful in the results.

Strongly convex function [19, 20]:

Suppose *l*_1_ is a positive real number. A function $\xi :B_{1}\subseteq R^{+}\to R$ is said to be strongly convex function with modulus *l*_1_, if

$$\begin{array}{c} \xi \left(ru_{1}+\left(1-r\right)u_{2}\right)\leq r\xi \left(u_{1}\right)+\left(1-r\right)\xi \left(u_{2}\right)-l_{1}r\left(1-r\right){\left(u_{1}-u_{2}\right)}^{2}, \end{array}$$

holds, ∀*u*_1_, *u*_2_ ∈ *B*_1_, and *r* ∈ [0, 1].

Super multiplicative function [21]:

A function *ξ*: $B_{1}\to R$ is called super multiplicative, if

$$\begin{array}{c} \xi \left(u_{1}u_{2}\right)\geq \xi \left(u_{1}\right)\xi \left(u_{2}\right),\;\forall \;u_{1},u_{2}\in B_{1}. \end{array}$$

Gamma function [22]:

Integral form of Gamma function is

$$\begin{array}{c} \Gamma \left(x\right)=\int_{0}^{\infty }e^{-k}k^{x-1}dk,\;where\;x,k>0. \end{array}$$

Riemann-Liouville (R-L) integrals [23]:

Suppose *ξ* ∈ *L*_1_[*c*, *d*], we can define *R* − *L* integrals $M_{c^{+}}^{\beta }\xi$ and $M_{d^{-}}^{\beta }\xi$ of order *β* > 0 as,

$$\begin{array}{c} M_{c^{+}}^{\beta }\xi \left(y\right)=\frac{1}{\Gamma (\beta )}\int_{c}^{y}{\left(y-k\right)}^{\beta -1}\xi \left(k\right)dk,\;with\;y>c; \end{array}$$

$$\begin{array}{c} M_{d^{-}}^{\beta }\xi \left(y\right)=\frac{1}{\Gamma (\beta )}\int_{y}^{d}{\left(k-y\right)}^{\beta -1}\xi \left(k\right)dk,\;with\;y<d. \end{array}$$

Caputo fractional derivatives (CFD) [24–26]:

Suppose *AC*^*n*^[*a*, *b*] be the space of functions that have nth derivatives absolutely continuous, *ξ* ∈ *AC*^*n*^[*a*, *b*], where *n* = [*β*] + 1, then we can define *CFD* Mca+β(ξ) and Mcb−β(ξ) of order *β* > 0 as,

Mca+βξ(y)=1Γ(n-β)∫ay(y-k)n-β-1ξn(k)dk,withy>a;

Mcb−βξ(y)=(-1)nΓ(n-β)∫yb(k-y)n-β-1ξn(k)dk,withy<b.

## 3 Main results

The novel class of convex functions named as strongly modified (*p*, *h*)-convex functions is introduced in this section.

Let $h:C_{1}\subseteq R\to R$ be non-zero, non-negative function, and *l*_1_ be a positive real number. A function $\xi :B_{1}\subseteq R\to R$ is said to be strongly modified (*p*, *h*)-convex function with modulus *l*_1_, if

$$\begin{array}{c} \xi ({\left(r{u_{1}}^{p}+\left(1-r\right){u_{2}}^{p}\right)}^{1/p})\leq h\left(r\right)\xi \left(u_{1}\right)+\left(1-h\left(r\right)\right)\xi \left(u_{2}\right)-l_{1}r\left(1-r\right){\left({u_{1}}^{p}-{u_{2}}^{p}\right)}^{2}, \end{array}$$ (1)

holds, ∀*u*_1_, *u*_2_ ∈ *B*_1_, *p* ≥ 1, and *r* ∈ (0, 1).

**Remark 1** *(a) By choosing l*_1_ = 0, *p* = 1, *and h*(*r*) = *r in inequality* (1), *one obtains the convex function*.

*(b) If we choose l*_1_ = 0, *and h*(*r*) = *r in inequality* (1), *one obtains the p-convex function*.

*(c) By choosing l*_1_ = 0, *and p* = 1 *in inequality* (1), *one obtains the modified h-convex function* (*see* [27]).

*(d) If we put l*_1_ = 0 *in inequality* (1), *one obtains the modified* (*p*, *h*)-*convex function*.

The validity of this novel concept of convexity is presented in the following example:

**Example 2.1** *Suppose u*_1_, *u*_2_ ∈ [1, ∞), *r* ∈ (0, 1), *p* ≥ 1, *l*_1_ > 0 *and h*(*r*) = *r*^5^, *then the function ξ*(*u*) = *u*^4^ *is strongly modified* (*p*, *h*)-*convex function*.

*Particularly, if we choose u*_1_, *u*_2_ ∈ [1, ∞) *with u*_1_ < *u*_2_, *r* = 1/2, *p* = 2 *and l*_1_ = 1/2, *in inequality* (1), *we get*

$$\begin{array}{c} (\frac{u_{1}^{2}+u_{2}^{2}}{2}{)}^{2}\leq \frac{u_{1}^{4}}{32}+\frac{31u_{2}^{4}}{32}-\frac{u_{1}^{2}}{8}-\frac{u_{2}^{2}}{8}+\frac{u_{1}u_{2}}{4}. \end{array}$$ (2)

**Fig 1:**
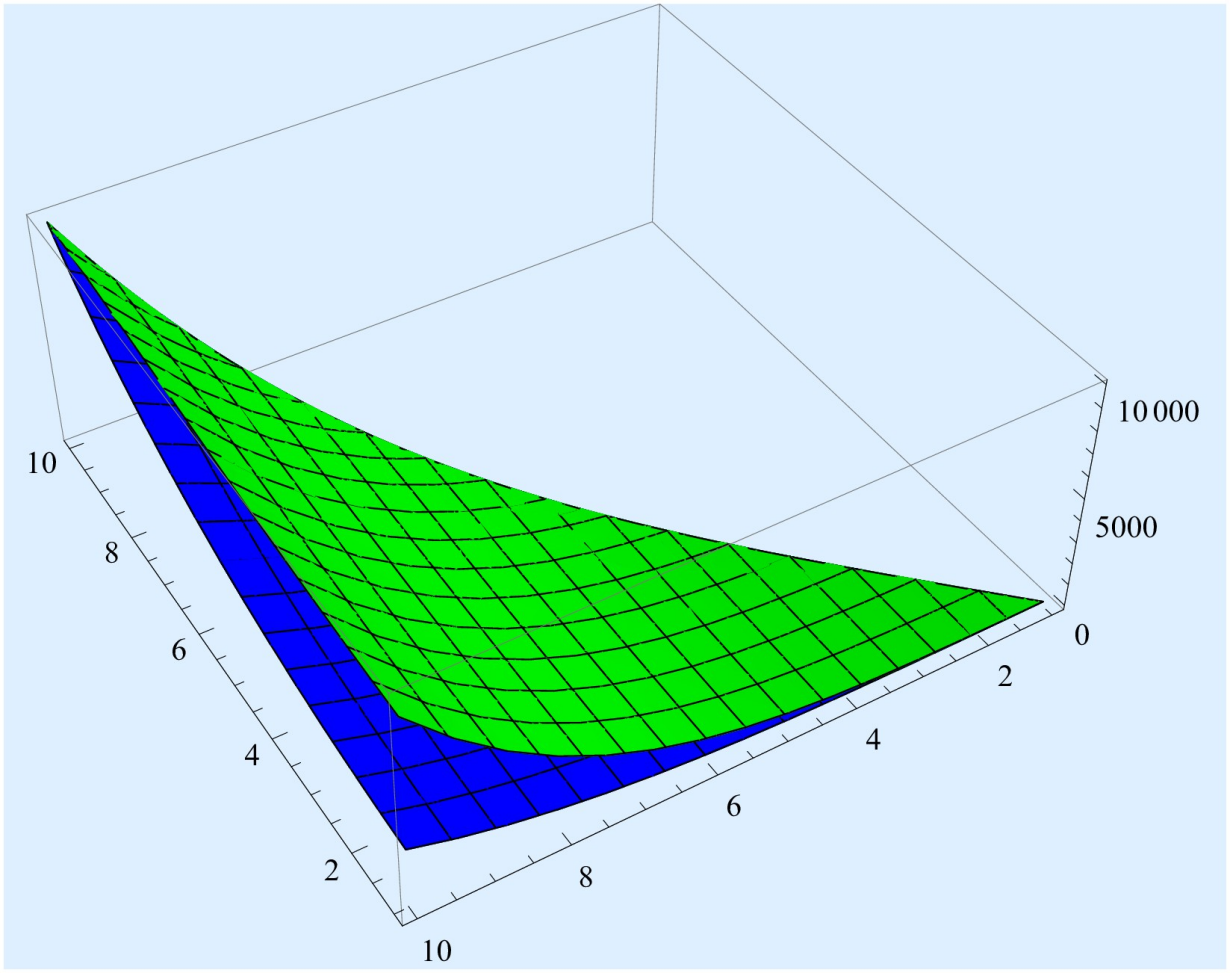
The graphical presentations of inequality (2).

Fig 1 *presents the validity of inequality* (2).

Green and Blue colours represent the right hand side and the left hand side of inequality (2) respectively.

**Example 2.2** *Consider u*_1_, *u*_2_ ∈ [1, ∞), *r* ∈ (0, 1), *then the function ξ*(*u*) = *u*^3^ *is convex function but not strongly modified* (*p*, *h*)-*convex function for h*(*r*) = *r*^5^, *p* ≥ 1, *and l*_1_ > 0.

*Particularly, if we choose u*_1_, *u*_2_ ∈ [1, ∞) *with u*_1_ < *u*_2_, *r* = 1/2, *p* = 2 *and l*_1_ = 1/2, *in inequality* (1), *we get*

$$\begin{array}{c} (\frac{u_{1}^{2}+u_{2}^{2}}{2}{)}^{3/2}≰\frac{u_{1}^{3}}{32}+\frac{31u_{2}^{3}}{32}-\frac{u_{1}^{4}}{8}-\frac{u_{2}^{4}}{8}+\frac{u_{1}^{2}u_{2}^{2}}{4}. \end{array}$$ (3)

**Fig 2:**
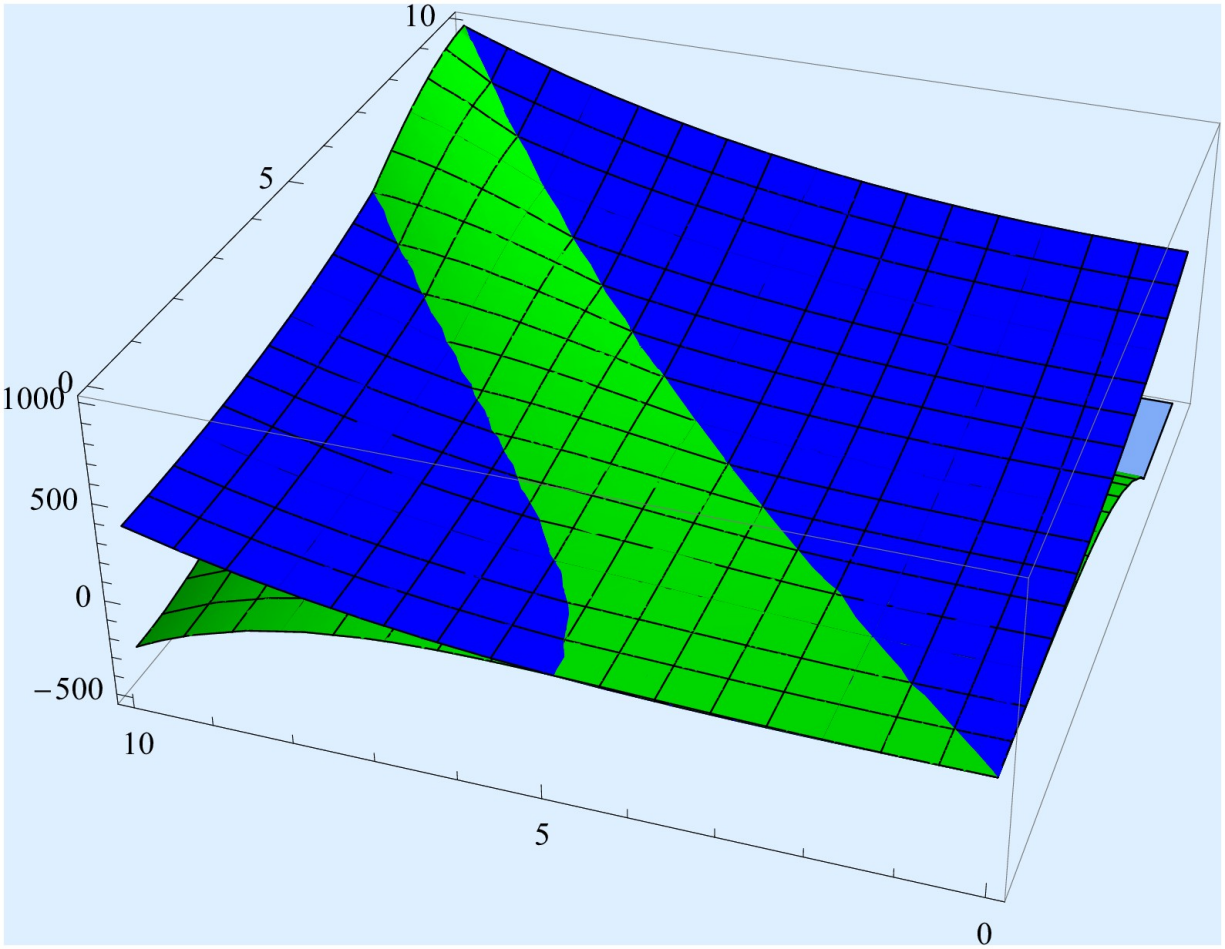
The graphical presentations of inequality (3).

Fig 2 *presents the graph of inequality* (3).

Green and Blue colours represent the right hand side and the left hand side of inequality (3) respectively.

Now, some basic properties of strongly modified (*p*, *h*)-convex functions are proved.

**Lemma 2.1** *Let ξ and* Φ *be strongly modified* (*p*, *h*)-*convex functions, then their sum is also strongly modified* (*p*, *h*)-*convex function*.

**Proof 1** *For u*_1_, *u*_2_ ∈ *B*_1_, *p* ≥ 1, *l*_1_ > 0 *and r* ∈ (0, 1), *we have*

$$\begin{array}{c} \begin{array}{cc} & \left(\xi +\Phi \right)({\left(ru_{1}^{p}+\left(1-r\right)u_{2}^{p}\right)}^{1/p})\\ & =\xi ({\left(ru_{1}^{p}+\left(1-r\right)u_{2}^{p}\right)}^{1/p})+\Phi ({\left(ru_{1}^{p}+\left(1-r\right)u_{2}^{p}\right)}^{1/p}). \end{array} \end{array}$$

*Since ξ and* Φ *are strongly modified* (*p*, *h*)-*convex functions*,

$$\begin{array}{c} \begin{array}{cc} & \left(\xi +\Phi \right)({\left(ru_{1}^{p}+\left(1-r\right)u_{2}^{p}\right)}^{1/p})\\ & \leq h\left(r\right)\xi \left(u_{1}\right)+\left(1-h\left(r\right)\right)\xi \left(u_{2}\right)-l_{1}r\left(1-r\right){\left(u_{1}^{p}-u_{2}^{p}\right)}^{2}\\ & +h\left(r\right)\Phi \left(u_{1}\right)+\left(1-h\left(r\right)\right)\Phi \left(u_{2}\right)-l_{1}r\left(1-r\right){\left(u_{1}^{p}-u_{2}^{p}\right)}^{2}\\ & =h\left(r\right)\left(\xi +\Phi \right)\left(u_{1}\right)+\left(1-h\left(r\right)\right)\left(\xi +\Phi \right)\left(u_{2}\right))-\left(l_{1}r\left(1-r\right){\left(u_{1}^{p}-u_{2}^{p}\right)}^{2}\right). \end{array} \end{array}$$

**Lemma 2.2** *Let ξ be strongly modified* (*p*, *h*)-*convex function, then for scalar n* > 0, *nξ is also strongly modified* (*p*, *h*)-*convex function*.

**Proof 2** *For u*_1_, *u*_2_ ∈ *B*_1_, *p* ≥ 1, *l*_1_ > 0 *and r* ∈ (0, 1), *we have*

$$\begin{array}{c} \begin{array}{cc} & n\xi ({\left(ru_{1}^{p}+\left(1-r\right)u_{2}^{p}\right)}^{1/p})\\ & \leq n(h\left(r\right)\xi \left(u_{1}\right)+\left(1-h\left(r\right)\right)\xi \left(u_{2}\right)-l_{1}r\left(1-r\right){\left(u_{1}^{p}-u_{2}^{p}\right)}^{2})\\ & =h\left(r\right)n\xi \left(u_{1}\right)+\left(1-h\left(r\right)\right)n\xi \left(u_{2}\right)-nl_{1}r\left(1-r\right){\left(u_{1}^{p}-u_{2}^{p}\right)}^{2}. \end{array} \end{array}$$

**Lemma 2.3** *Let h*_1_, *h*_2_ *be two non-zero, non-negative functions on* $C_{1}\subseteq R$ *such that h*_2_(*r*) ≤ *h*_1_(*r*). *If* $\xi :B_{1}\subseteq R\to R$ *is strongly modified h*_2_-*convex function, then ξ is also strongly modified h*_1_-*convex function*.

**Proof 3** *For u*_1_, *u*_2_ ∈ *B*_1_, *p* ≥ 1, *l*_1_ > 0 *and r* ∈ (0, 1), *we have*

$$\begin{array}{c} \begin{array}{cc} & \xi ({\left(ru_{1}^{p}+\left(1-r\right)u_{2}^{p}\right)}^{1/p})\\ & \leq h_{2}\left(r\right)\xi \left(u_{1}\right)+\left(1-h_{2}\left(r\right)\right)\xi \left(u_{2}\right)-l_{1}r\left(1-r\right){\left(u_{1}^{p}-u_{2}^{p}\right)}^{2}\\ & \leq h_{1}\left(r\right)\xi \left(u_{1}\right)+\left(1-h_{1}\left(r\right)\right)\xi \left(u_{2}\right)-l_{1}r\left(1-r\right){\left(u_{1}^{p}-u_{2}^{p}\right)}^{2}. \end{array} \end{array}$$

**Remark 2** *Let h*_1_, *h*_2_ *be two non-zero, non-negative functions on* $C_{1}\subseteq R$ *such that h*_1_(*r*) ≤ *h*_2_(*r*) *and if* $\xi :B_{1}\subseteq R\to R$ *is strongly modified h*_1_-*convex function, then ξ is also strongly modified h*_2_-*convex function*.

**Lemma 2.4** *Let* $\xi_{s}:B_{1}\subseteq R\to R$ *be strongly modified* (*p*, *h*)-*convex functions for* s∈N *and* $\sum_{s=1}^{d}n_{s}=1$, *then their linear combination*

$$\begin{array}{c} W\left(l\right)=\sum_{s=1}^{d}n_{s}\xi_{s}\left(l\right), \end{array}$$ (4)

*for all l* ∈ *B*_1_ *is also strongly modified* (*p*, *h*)-*convex function*.

**Proof 4** *For u*_1_, *u*_2_ ∈ *B*_1_, *p* ≥ 1, *l*_1_ > 0, *r* ∈ (0, 1) *and* $l={\left(ru_{1}^{p}+\left(1-r\right)u_{2}^{p}\right)}^{1/p}$ *in* (4), *we get*

$$\begin{array}{ccc} & \; & W{\left(ru_{1}^{p}+\left(1-r\right)u_{2}^{p}\right)}^{1/p}=\sum_{s=1}^{d}n_{s}(\xi_{s}{\left(ru_{1}^{p}+\left(1-r\right)u_{2}^{p}\right)}^{1/p}\\ & \; & \leq h\left(r\right)(\sum_{s=1}^{d}n_{s}\left(\xi_{s}\left(u_{1}\right)\right))+\left(1-h\left(r\right)\right)(\sum_{s=1}^{d}n_{s}(\xi_{s}\left(u_{2}\right)))-\sum_{s=1}^{d}n_{s}l_{1}r\left(1-r\right){\left(u_{1}^{p}-u_{2}^{p}\right)}^{2}\\ & \; & =h\left(u\right)W\left(u_{1}\right)+\left(1-h\left(u\right)\right)W\left(u_{2}\right)-l_{1}r\left(1-r\right){\left({u_{1}}^{p}-{u_{2}}^{p}\right)}^{2}. \end{array}$$

**Lemma 2.5** *Let* $\left\{\xi_{s}:B_{1}\subseteq R\to R;s\in N\right\}$ *be non-empty collection of strongly modified* (*p*, *h*)-*convex functions such that for all l* ∈ *B*_1_, ${\text{sup}}_{s\in N}\xi_{s}\left(l\right)$ *exists in* R. *The function* $\xi :B_{1}\subseteq R\to R$ *defined by* $\xi \left(l\right)={\text{sup}}_{s\in N}\xi_{s}\left(l\right)$ *for all l* ∈ *B*_1_ *is also strongly modified* (*p*, *h*)-*convex function*.

**Proof 5** *For u*_1_, *u*_2_ ∈ *B*_1_, *p* ≥ 1, *l*_1_ > 0 *and r* ∈ (0, 1), *we have*

$$\begin{array}{c} \xi \left(l\right)=\underset{s\in N}{\text{sup}}\;\xi_{s}\left(l\right). \end{array}$$ (5)

*By choosing* $l={\left(ru_{1}^{p}+\left(1-r\right)u_{2}^{p}\right)}^{1/p}$ *in inequality* (5), *we get*

$$\begin{array}{ccc} & \; & \xi {\left(ru_{1}^{p}+\left(1-r\right)u_{2}^{p}\right)}^{1/p}=\underset{s\in N}{\text{sup}}\;\xi_{s}{\left(ru_{1}^{p}+\left(1-r\right)u_{2}^{p}\right)}^{1/p}\\ & \; & \leq h\left(r\right)(\underset{s\in N}{\text{sup}}\;\xi \left(u_{1}\right))+\left(1-h\left(r\right)\right)(\underset{s\in N}{\text{sup}}\;\xi \left(u_{2}\right))-l_{1}r\left(1-r\right){\left(u_{1}^{p}-u_{2}^{p}\right)}^{2}\\ & \; & =h\left(r\right)\xi \left(u_{1}\right)+\left(1-h\left(r\right)\right)\xi \left(u_{2}\right)-l_{1}r\left(1-r\right){\left(u_{1}^{p}-u_{2}^{p}\right)}^{2}. \end{array}$$

**Lemma 2.6** *Let ξ* *be strongly modified* (*p*, *h*)-*convex function, then* $\xi {\left(u_{1}^{p}+u_{2}^{p}-w^{p}\right)}^{1/p}\leq \xi \left(u_{1}\right)+\xi \left(u_{2}\right)-\xi \left(w\right)$ *for all w* ∈ *B*_1_, *p* ≥ 1 *and r* ∈ [0, 1]. *Where*, $w^{p}=ru_{1}^{p}+\left(1-r\right)u_{2}^{p}$.

**Proof 6** *For w* ∈ *B*_1_ *and r* ∈ (0, 1), *we have*

$$\begin{array}{c} \begin{array}{cc} & \xi {\left({u_{1}}^{p}+{u_{2}}^{p}-w^{p}\right)}^{1/p}=\xi {\left(\left(1-r\right){u_{1}}^{p}+r{u_{2}}^{p}\right)}^{1/p}\\ & \leq \left(1-h\left(r\right)\right)\xi \left(u_{1}\right)+h\left(r\right)\xi \left(u_{2}\right)-l_{1}r\left(1-r\right){\left({u_{2}}^{p}-{u_{1}}^{p}\right)}^{2}\\ & =\left(1-h\left(r\right)\right)\xi \left(u_{1}\right)+\xi \left(u_{2}\right)-\xi \left(u_{2}\right)+h\left(r\right)\xi \left(u_{2}\right)+l_{1}r\left(1-r\right){\left(u_{1}^{p}-u_{2}^{p}\right)}^{2}\\ & \leq \xi \left(u_{1}\right)+\xi \left(u_{2}\right)-(h\left(r\right)\xi \left(u_{1}\right)+\left(1-h\left(r\right)\right)\xi \left(u_{2}\right)-l_{1}r\left(1-r\right){\left(u_{1}^{p}-u_{2}^{p}\right)}^{2})\\ & =\xi \left(u_{1}\right)+\xi \left(u_{2}\right)-\xi \left(w\right). \end{array} \end{array}$$

## 4 Schur inequality

The next theorem presents the Schur inequality for the strongly modified (*p*, *h*)-convex function.

**Theorem 1** *Let* $\xi :B_{1}\subseteq R\to R$ *be strongly modified* (*p*, *h*)-*convex function and* $h:C_{1}\subseteq R\to R$ *be non-zero, non-negative, super multiplicative function, then for u*_1_, *u*_2_, *u*_3_ ∈ *B*_1_ *such that u*_1_ < *u*_2_ < *u*_3_, *u*_3_ − *u*_1_, *u*_3_ − *u*_2_, *u*_2_ − *u*_1_ ∈ *B*_1_ *and r* ∈ (0, 1), *we have*

$$\begin{array}{ccc} \xi \left(u_{2}\right)\cdot h\left(u_{3}^{p}-u_{1}^{p}\right) & \; & \leq h\left(u_{3}^{p}-u_{1}^{p}\right)\xi \left(u_{1}\right)+(h\left(u_{3}^{p}-u_{1}^{p}\right)-h\left(u_{3}^{p}-u_{2}^{p}\right))\xi \left(u_{3}\right)\\ & \; & -h\left(u_{3}^{p}-u_{1}^{p}\right)l_{1}\left(u_{3}^{p}-u_{2}^{p}\right)\left(u_{2}^{p}-u_{1}^{p}\right). \end{array}$$ (6)

**Proof 7** *Let u*_1_, *u*_2_, *u*_3_ ∈ *B*_1_ *be such that* $(\frac{u_{3}-u_{2}}{u_{3}-u_{1}})\in \left(0,1\right)$ *and* $(\frac{u_{2}-u_{1}}{u_{3}-u_{1}})\in \left(0,1\right)$, *then we have*

$$\begin{array}{c} h\left(u_{3}-u_{2}\right)=h(\frac{u_{3}-u_{2}}{u_{3}-u_{1}}\times \left(u_{3}-u_{1}\right))\leq h(\frac{u_{3}-u_{2}}{u_{3}-u_{1}})\times h\left(u_{3}-u_{1}\right). \end{array}$$

*Suppose h*(*u*_3_ − *u*_2_) > 0, *then by definition of ξ*, *we get*

$$\begin{array}{c} \begin{array}{c} \xi {\left(rs^{p}+\left(1-r\right)w^{p}\right)}^{1/p}\leq h\left(r\right)\xi \left(s\right)+\left(1-h\left(r\right)\right)\xi \left(w\right)-l_{1}r\left(1-u_{1}\right){\left(s^{p}-w^{p}\right)}^{2}. \end{array} \end{array}$$ (7)

*By choosing* $\frac{\left(u_{3}^{p}-u_{2}^{p}\right)}{\left(u_{3}^{p}-u_{1}^{p}\right)}=r$ , *s* = *u*_1_ *and w* = *u*_3_ *in* (7), *we get*

$$\begin{array}{c} \xi \left(u_{2}\right)\leq h(\frac{u_{3}^{p}-u_{2}^{p}}{u_{3}^{p}-u_{1}^{p}})\xi \left(u_{1}\right)+(1-h(\frac{u_{3}^{p}-u_{2}^{p}}{u_{3}^{p}-u_{1}^{p}}))\xi \left(u_{3}\right)-l_{1}\frac{\left(u_{3}^{p}-u_{2}^{p}\right)\left(u_{2}^{p}-u_{1}^{p}\right)}{{\left(u_{1}^{p}-u_{3}^{p}\right)}^{2}}{\left(u_{1}^{p}-u_{3}^{p}\right)}^{2}. \end{array}$$

$$\begin{array}{c} \begin{array}{cc} & \xi \left(u_{2}\right)\leq (\frac{h(u_{3}^{p}-u_{2}^{p})}{h(u_{3}^{p}-u_{1}^{p})})\xi \left(u_{1}\right)+(1-(\frac{h(u_{3}^{p}-u_{2}^{p})}{h(u_{3}^{p}-u_{1}^{p})}))\xi \left(u_{3}\right)-l_{1}\left(u_{3}^{p}-u_{2}^{p}\right)\left(u_{2}^{p}-u_{1}^{p}\right). \end{array} \end{array}$$

$$\begin{array}{ccc} \Rightarrow \xi \left(u_{2}\right)\times h\left(u_{3}^{p}-u_{1}^{p}\right) & \; & \leq h(u_{3}^{p}-u_{2}^{p})\xi \left(u_{1}\right)+(h(u_{3}^{p}-u_{1}^{p})-h(u_{3}^{p}-u_{2}^{p}))\xi \left(u_{3}\right)\\ & \; & -h\left(u_{3}^{p}-u_{1}^{p}\right)\times l_{1}\left(u_{3}^{p}-u_{2}^{p}\right)\left(u_{2}^{p}-u_{1}^{p}\right). \end{array}$$

*Conversely*,

$$\begin{array}{ccc} \xi \left(u_{2}\right)\times h\left(u_{3}^{p}-u_{1}^{p}\right) & \; & \leq h(u_{3}^{p}-u_{2}^{p})\xi \left(u_{1}\right)+(h(u_{3}^{p}-u_{1}^{p})-h(u_{3}^{p}-u_{2}^{p}))\xi \left(u_{3}\right)\\ & \; & -h\left(u_{3}^{p}-u_{1}^{p}\right)\times l_{1}\left(u_{3}^{p}-u_{2}^{p}\right)\left(u_{2}^{p}-u_{1}^{p}\right). \end{array}$$

$$\begin{array}{c} \begin{array}{c} \Rightarrow \xi \left(u_{2}\right)\leq h(\frac{\left(u_{3}^{p}-u_{2}^{p}\right)}{\left(u_{3}^{p}-u_{1}^{p}\right)})\xi \left(u_{1}\right)+(1-h(\frac{u_{3}^{p}-u_{2}^{p}}{u_{3}^{p}-u_{1}^{p}}))\xi \left(u_{3}\right)-l_{1}\left(u_{3}^{p}-u_{2}^{p}\right)\left(u_{2}^{p}-u_{1}^{p}\right). \end{array} \end{array}$$ (8)

*By choosing*, $\frac{\left(u_{3}^{p}-u_{2}^{p}\right)}{\left(u_{3}^{p}-u_{1}^{p}\right)}=r$, *s* = *u*_1_ *and w* = *u*_3_ *in* (8), *we get*

$$\begin{array}{c} \begin{array}{c} \xi {\left(rs^{p}+\left(1-r\right)w^{p}\right)}^{1/p}\leq h\left(r\right)\xi \left(s\right)+\left(1-h\left(r\right)\right)\xi \left(w\right)-l_{1}r\left(1-u_{1}\right){\left(s^{p}-w^{p}\right)}^{2}. \end{array} \end{array}$$

*Thus, ξ is strongly modified* (*p*, *h*)-*convex function*.

The validity of schur inequality is presented in context of previously proved example below:

**Example 3.1** *Assuming, ξ*(*u*) = *u*^4^, *u*_1_ = 1, *u*_2_ = 2, *u*_3_ = 3, *p* = 2, $r=l_{1}=\frac{1}{2},$ *and h*(*r*) = *r*^5^ *in inequality* (6), *we get*

$$\begin{array}{ccc} {\left(u_{2}\right)}^{4}\times h\left(9-1\right)\leq & \; & h\left(9-1\right){\left(u_{1}\right)}^{4}+(h(9-1)-h(9-4)){\left(u_{3}\right)}^{4}\\ & \; & -h\left(9-1\right)l_{1}\left(u_{3}^{2}-u_{2}^{2}\right)\left(u_{2}^{2}-u_{1}^{2}\right). \end{array}$$ (9)

*By solving inequality* (9), *one gets*

$$\begin{array}{c} \Rightarrow 524,288\leq 2,188,091. \end{array}$$

## 5 Hermite-Hadamard inequalities

The Hermite-Hadamard Inequalities for this novel class of convex functions is given in next theorem.

**Theorem 2** *Suppose* $h:C_{1}\subseteq R\to R$ *is a non-zero, non-negative function and* $\xi :B_{1}=\left[u_{1},u_{2}\right]\subseteq R\to R$ *is a strongly modified* (*p*, *h*)-*convex function with u*_1_ < *u*_2_, *then*

$$\begin{array}{c} \begin{array}{cc} & \xi ((\frac{u_{1}^{p}+u_{2}^{p}}{2}{)}^{1/p})+\frac{l_{1}}{12}{\left(u_{1}^{p}-u_{2}^{p}\right)}^{2}\leq \frac{p}{\left(u_{2}^{p}-u_{1}^{p}\right)}\int_{u_{1}}^{u_{2}}\frac{\xi (s)}{s^{1-p}}ds\\ & \leq \xi \left(u_{1}\right)\int_{0}^{1}\left(h(r)\right)dr+\xi \left(u_{2}\right)\int_{0}^{1}\left(1-h(r)\right)dr-\frac{l_{1}}{6}{\left(u_{1}^{p}-u_{2}^{p}\right)}^{2}. \end{array} \end{array}$$

**Proof 8** *For s*, *w* ∈ *B*_1_, *p* ≥ 1, *l*_1_ > 0 *and n*_1_ ∈ [0, 1], *we have*

$$\begin{array}{c} \begin{array}{c} \xi ({\left(n_{1}s^{p}+\left(1-n_{1}\right)w^{p}\right)}^{1/p})\leq h\left(n_{1}\right)\xi \left(s\right)+\left(1-h\left(n_{1}\right)\right)\xi \left(w\right)-l_{1}n_{1}\left(1-n_{1}\right){\left(s^{p}-w^{p}\right)}^{2}. \end{array} \end{array}$$ (10)

*Put, n*_1_ = 1/2 *in* (10), *to get*

$$\begin{array}{c} \xi ((\frac{s^{p}+w^{p}}{2}{)}^{1/p})\leq h\left(1/2\right)\xi \left(s\right)+\left(1-h\left(1/2\right)\right)\xi \left(w\right)-\frac{l_{1}}{4}{\left(s^{p}-w^{p}\right)}^{2}. \end{array}$$ (11)

*Put*, $s^{p}=ru_{1}^{p}+\left(1-r\right)u_{2}^{p}$ *and* $w^{p}=\left(1-r\right)u_{1}^{p}+ru_{2}^{p}$ *in* (11), *to get*

$$\begin{array}{c} \begin{array}{cc} & \xi ((\frac{u_{1}^{p}+u_{2}^{p}}{2}{)}^{1/p})\leq h\left(1/2\right)\xi {\left(ru_{1}^{p}+\left(1-r\right)u_{2}^{p}\right)}^{1/p}\\ & +\left(1-h\left(1/2\right)\right)\xi {\left(\left(1-r\right)u_{1}^{p}+ru_{2}^{p}\right)}^{1/p}-\frac{l_{1}}{4}{\left(u_{1}^{p}-u_{2}^{p}\right)}^{2}{\left(2r-1\right)}^{2}. \end{array} \end{array}$$ (12)

*By integrating* (12) *with respect to r from* 0 *to* 1, *we get*

$$\begin{array}{c} \begin{array}{cc} & \int_{0}^{1}\xi ((\frac{u_{1}^{p}+u_{2}^{p}}{2}{)}^{1/p})dr\leq h\left(1/2\right)\int_{0}^{1}\xi {\left(ru_{1}^{p}+\left(1-r\right)u_{2}^{p}\right)}^{1/p}dr\\ & +\left(1-h\left(1/2\right)\right)\int_{0}^{1}\xi {\left(\left(1-r\right)u_{1}^{p}+ru_{2}^{p}\right)}^{1/p}dr-\frac{l_{1}}{4}{\left(u_{1}^{p}-u_{2}^{p}\right)}^{2}\int_{0}^{1}{\left(2r-1\right)}^{2}dr \end{array} \end{array}$$

$$\begin{array}{ccc} & \; & \Rightarrow \xi ((\frac{u_{1}^{p}+u_{2}^{p}}{2}{)}^{1/p})\leq h\left(1/2\right)\int_{0}^{1}\xi {\left(ru_{1}^{p}+\left(1-r\right)u_{2}^{p}\right)}^{1/p}dr\\ & \; & +\left(1-h\left(1/2\right)\right)\int_{0}^{1}\xi {\left(\left(1-r\right)u_{1}^{p}+ru_{2}^{p}\right)}^{1/p}dr-\frac{l_{1}}{12}{\left(u_{1}^{p}-u_{2}^{p}\right)}^{2}. \end{array}$$ (13)

*Put*, $s^{p}=ru_{1}^{p}+\left(1-r\right)u_{2}^{p}$ *in first integral of* (13), *and* $s^{p}=\left(1-r\right)u_{1}^{p}+ru_{2}^{p}$ *in second integral of* (13), *to get*

$$\begin{array}{ccc} & \; & \xi ({\left(\frac{u_{1}^{p}+u_{2}^{p}}{2}\right)}^{1/p})\leq h\left(1/2\right)\frac{p}{\left(u_{2}^{p}-u_{1}^{p}\right)}\int_{u_{1}}^{u_{2}}\frac{\xi (s)}{s^{1-p}}ds\\ & \; & +\left(1-h\left(1/2\right)\right)\frac{p}{\left(u_{2}^{p}-u_{1}^{p}\right)}\int_{u_{1}}^{u_{2}}\frac{\xi (s)}{s^{1-p}}ds-\frac{l_{1}}{12}{\left(u_{1}^{p}-u_{2}^{p}\right)}^{2}. \end{array}$$

$$\begin{array}{c} \Rightarrow \xi ((\frac{u_{1}^{p}+u_{2}^{p}}{2}{)}^{1/p})+\frac{l_{1}}{12}{\left(u_{1}^{p}-u_{2}^{p}\right)}^{2}\leq \frac{p}{\left(u_{2}^{p}-u_{1}^{p}\right)}\int_{u_{1}}^{u_{2}}\frac{\xi (s)}{s^{1-p}}ds. \end{array}$$ (14)

*Put* $s^{p}=ru_{1}^{p}+\left(1-r\right)u_{2}^{p},$ *on right hand side of* (14) *to get*

$$\begin{array}{c} \begin{array}{cc} & \frac{p}{\left(u_{2}^{p}-u_{1}^{p}\right)}\int_{u_{1}}^{u_{2}}\frac{\xi (s)}{s^{1-p}}ds=\int_{0}^{1}\xi \left(ru_{1}^{p}+\left(1-r\right)u_{2}^{p}\right)dr\\ & \leq \int_{0}^{1}\left(h\left(r\right)\right)\xi \left(u_{1}\right)dr+\int_{0}^{1}\left(1-h\left(r\right)\right)\xi \left(u_{2}\right)dr-\int_{0}^{1}\left(l_{1}r\left(1-r\right){\left(u_{1}^{p}-u_{2}^{p}\right)}^{2}\right)dr. \end{array} \end{array}$$

$$\begin{array}{c} \Rightarrow \frac{p}{\left(u_{2}^{p}-u_{1}^{p}\right)}\int_{u_{1}}^{u_{2}}\frac{\xi (s)}{s^{1-p}}ds\leq \xi \left(u_{1}\right)\int_{0}^{1}\left(h(r)\right)dr+\xi \left(u_{2}\right)\int_{0}^{1}\left(1-h(r)\right)dr-\frac{l_{1}}{6}{\left(u_{1}^{p}-u_{2}^{p}\right)}^{2}. \end{array}$$ (15)

*From* (14) *and* (15), *we get*

$$\begin{array}{c} \begin{array}{cc} & \xi ((\frac{u_{1}^{p}+u_{2}^{p}}{2}{)}^{1/p})+\frac{l_{1}}{12}{\left(u_{1}^{p}-u_{2}^{p}\right)}^{2}\leq \frac{p}{\left(u_{2}^{p}-u_{1}^{p}\right)}\int_{u_{1}}^{u_{2}}\frac{\xi (s)}{s^{1-p}}ds\\ & \leq \xi \left(u_{1}\right)\int_{0}^{1}\left(h(r)\right)dr+\xi \left(u_{2}\right)\int_{0}^{1}\left(1-h(r)\right)dr-\frac{l_{1}}{6}{\left(u_{1}^{p}-u_{2}^{p}\right)}^{2}. \end{array} \end{array}$$

The following remark presents that the Theorem 2 generalizes the results that already exist in literature.

**Remark 3** *(a) Assume h*(*r*) = *r in Theorem 2, to get Theorem* 2.1 *of* [28].

*(b) Assume h*(*r*) = *r and p* = 1 *in Theorem 2, to obtain Theorem* 6 *of* [29].

*(c) Assume l*_1_ = 0 *in Theorem 2, to get Theorem* 3 *of* [18].

*(d) Assume l*_1_ = 0 *and h*(*r*) = *r in Theorem 2, to obtain H-H inequalities for convex function* (*see* [5]).

The following example validate the Theorem 2.

**Example 4.1** *Assuming ξ*(*u*) = *u*^4^, *u*_1_ = 1, *u*_2_ = 2, *p* = 2, $r=l_{1}=\frac{1}{2}$ *and h*(*r*) = *r*^5^ *in inequality* (6), *we get*

$$\begin{array}{ccc} \xi (\frac{1+4}{2}{)}^{1/2}+\frac{1}{24}{\left(1-4\right)}^{2} & \; & \leq \frac{2}{\left(4-1\right)}\int_{1}^{2}\frac{\xi (s)}{s^{1-2}}ds\\ & \; & \leq \xi \left(1\right)\int_{0}^{1}h(r)dr+\xi \left(2\right)\int_{0}^{1}h(r)dr-\frac{1}{12}{\left(1-4\right)}^{2}.\\ \Rightarrow 2.625\leq 7\leq 12.75. \end{array}$$

The next theorem presents the H-H inequalities for this novel concept of convexity by utilizing Riemann-Liouville integrals.

**Theorem 3** *Let* ξ:[0,1]→R *be a strongly modified* (*p*, *h*)-*convex function with u*_1_ < *u*_2_ *for any u*_1_, *u*_2_ ∈ [0, 1], *then*

$$\begin{array}{c} \begin{array}{cc} & \frac{1}{\beta }(\xi (\frac{u_{1}^{p}+u_{2}^{p}}{2}{)}^{1/p})\leq \frac{ps^{1-p}\Gamma \left(\beta \right)}{{\left(u_{2}^{p}-u_{1}^{p}\right)}^{\beta }}(\left(1-h\left(1/2\right)\right)M_{u_{1}^{+}}^{\beta }\xi \left(u_{2}\right)+h\left(1/2\right)M_{u_{2}^{-}}^{\beta }\xi \left(u_{1}\right))\\ & -\frac{l_{1}}{4}\;{\left(u_{1}^{p}-u_{2}^{p}\right)}^{2}\;\frac{\beta^{2}-\beta +2}{\beta^{3}+3\beta^{2}+2\beta }, \end{array} \end{array}$$

*and*

$$\begin{array}{c} \frac{ps^{1-p}\Gamma \left(\beta \right)}{{\left(u_{2}^{p}-u_{1}^{p}\right)}^{\beta }}(M_{u_{1}^{+}}^{\beta }\xi \left(u_{2}\right)+M_{u_{2}^{-}}^{\beta }\xi \left(u_{1}\right))\leq \frac{\xi \left(u_{1}\right)+\xi \left(u_{2}\right)}{\beta }-\frac{2l_{1}{\left(u_{1}^{p}-u_{2}^{p}\right)}^{2}}{\beta^{2}+3\beta +2}. \end{array}$$

**Proof 9** *Since ξ is strongly modified* (*p*, *h*)-*convex function, therefore*

$$\begin{array}{c} \xi ({\left(\left(1-r\right)s^{p}+rw^{p}\right)}^{1/p})\leq \left(1-h\left(r\right)\right)\xi \left(s\right)+h\left(r\right)\xi \left(w\right))-l_{1}r\left(1-r\right){\left(s^{p}-w^{p}\right)}^{2}. \end{array}$$ (16)

*By putting r* = 1/2 *in* (16), *we get*

$$\begin{array}{c} \xi ((\frac{s^{p}+w^{p}}{2}{)}^{1/p})\leq \left(1-h\left(1/2\right)\right)\xi \left(s\right)+h\left(1/2\right)\xi \left(w\right)-\frac{l_{1}}{4}{\left(s^{p}-w^{p}\right)}^{2}. \end{array}$$ (17)

*Assuming*, $s^{p}={\left(ru_{1}^{p}+\left(1-r\right)u_{2}^{p}\right)}^{1/p}$ *and* $l^{p}={\left(\left(1-r\right)u_{1}^{p}+ru_{2}^{p}\right)}^{1/p}$ *in* (17), *to get*

$$\begin{array}{ccc} & \; & \xi ((\frac{u_{1}^{p}+u_{2}^{p}}{2}{)}^{1/p})\leq \left(1-h\left(1/2\right)\right)\xi {\left(ru_{1}^{p}+\left(1-r\right)u_{2}^{p}\right)}^{1/p}\\ & \; & +h\left(1/2\right)\xi {\left(\left(1-r\right)u_{1}^{p}+ru_{2}^{p}\right)}^{1/p}-\frac{l_{1}}{4}{\left(u_{1}^{p}-u_{2}^{p}\right)}^{2}{\left(2r-1\right)}^{2}. \end{array}$$ (18)

*Multiply* (18) *by r*^*β*^ − 1 *and then integrate from* 0 *to* 1 *with respect to r, to get*

$$\begin{array}{c} \begin{array}{cc} & \int_{0}^{1}({\left(r\right)}^{\beta -1}\times \xi (\frac{u_{1}^{p}+u_{2}^{p}}{2}{)}^{1/p})dr\leq \left(1-h\left(1/2\right)\right)\int_{0}^{1}\left({\left(r\right)}^{\beta -1}\times \xi {\left(ru_{1}^{p}+\left(1-r\right)u_{2}^{p}\right)}^{1/p}\right)dr\\ & +h\left(1/2\right)\int_{0}^{1}\left({\left(r\right)}^{\beta -1}\times \xi {\left(\left(1-r\right)u_{1}^{p}+ru_{2}^{p}\right)}^{1/p}\right)dr-\frac{l_{1}}{4}{\left(u_{1}^{p}-u_{2}^{p}\right)}^{2}\int_{0}^{1}\left({\left(r\right)}^{\beta -1}\times {\left(2r-1\right)}^{2}\right)dr. \end{array} \end{array}$$

$$\begin{array}{c} \begin{array}{cc} & \Rightarrow \frac{1}{\beta }(\xi (\frac{u_{1}^{p}+u_{2}^{p}}{2}{)}^{1/p})\leq \left(1-h\left(1/2\right)\right)\int_{0}^{1}\left({\left(r\right)}^{\beta -1}\times \xi {\left(ru_{1}^{p}+\left(1-r\right)u_{2}^{p}\right)}^{1/p}\right)dr\\ & h\left(1/2\right)\int_{0}^{1}\left({\left(r\right)}^{\beta -1}\times \xi {\left(\left(1-r\right)u_{1}^{p}+ru_{2}^{p}\right)}^{1/p}\right)dr-\frac{l_{1}}{4}\times {\left(u_{1}^{p}-u_{2}^{p}\right)}^{2}\times \frac{\beta^{2}-\beta +2}{\beta^{3}+3\beta^{2}+2\beta }. \end{array} \end{array}$$ (19)

*Use* $s^{p}=\left(ru_{1}^{p}+\left(1-r\right)u_{2}^{p}\right),$ *in first integral of* (19) *and* $s^{p}=\left(\left(1-r\right)u_{1}^{p}+ru_{2}^{p}\right),$ *in second integral of* (19), *to get*

$$\begin{array}{c} \begin{array}{cc} & \frac{1}{\beta }(\xi (\frac{u_{1}^{p}+u_{2}^{p}}{2}{)}^{1/p})\leq \left(1-h\left(1/2\right)\right)\int_{u_{1}}^{u_{2}}((\frac{u_{2}^{p}-s^{p}}{u_{2}^{p}-u_{1}^{p}}{)}^{\beta -1}\times \frac{ps^{1-p}\xi \left(s\right)}{u_{2}^{p}-u_{1}^{p}})ds\\ & +h\left(1/2\right)\int_{u_{1}}^{u_{2}}((\frac{s^{p}-u_{1}^{p}}{u_{2}^{p}-u_{1}^{p}}{)}^{\beta -1}\times \frac{ps^{1-p}\xi \left(s\right)}{u_{2}^{p}-u_{1}^{p}})ds-\frac{l_{1}}{4}\times {\left(u_{1}^{p}-u_{2}^{p}\right)}^{2}\times \frac{\beta^{2}-\beta +2}{\beta^{3}+3\beta^{2}+2\beta }. \end{array} \end{array}$$ (20)

*Since*,

$$\begin{array}{c} \begin{array}{cc} & M_{u_{1}^{+}}^{\beta }\xi \left(u_{2}\right)=\frac{1}{\Gamma (\beta )}\int_{u_{1}}^{u_{2}}{\left(u_{2}-s\right)}^{\beta -1}\xi \left(s\right)ds,\\ & M_{u_{2}^{-}}^{\beta }\xi \left(u_{1}\right)=\frac{1}{\Gamma (\beta )}\int_{u_{1}}^{u_{2}}{\left(s-u_{1}\right)}^{\beta -1}\xi \left(s\right)ds. \end{array} \end{array}$$

*Therefore*, (20) *become*

$$\begin{array}{ccc} & \; & \frac{1}{\beta }(\xi (\frac{u_{1}^{p}+u_{2}^{p}}{2}{)}^{1/p})\leq \frac{ps^{1-p}\Gamma \left(\beta \right)}{{\left(u_{2}^{p}-u_{1}^{p}\right)}^{\beta }}(\left(1-h\left(1/2\right)\right)M_{u_{1}^{+}}^{\beta }\xi \left(u_{2}\right)+h\left(1/2\right)M_{u_{2}^{-}}^{\beta }\xi \left(u_{1}\right))\\ & \; & -\frac{l_{1}}{4}\times {\left(u_{1}^{p}-u_{2}^{p}\right)}^{2}\times \frac{\beta^{2}-\beta +2}{\beta^{3}+3\beta^{2}+2\beta }. \end{array}$$ (21)

*Also, ξ is strongly modified* (*p*, *h*)-*convex function, therefore*

$$\begin{array}{c} \xi {\left(ru_{1}^{p}+\left(1-r\right)u_{2}^{p}\right)}^{1/p}\leq h\left(r\right)\xi \left(u_{1}\right)+(1-h\left(r\right)\xi \left(u_{2}\right)-l_{1}r\left(1-r\right){\left(u_{1}^{p}-u_{2}^{p}\right)}^{2}, \end{array}$$ (22)

*and*

$$\begin{array}{c} \xi {\left(\left(1-r\right)u_{1}^{p}+ru_{2}^{p}\right)}^{1/p}\leq \left(1-h\left(r\right)\right)\xi \left(u_{1}\right)+h\left(r\right)\xi \left(u_{2}\right)-l_{1}r\left(1-r\right){\left(u_{1}^{p}-u_{2}^{p}\right)}^{2}. \end{array}$$ (23)

*By adding* (22) *and* (23), *we get*

$$\begin{array}{ccc} & \; & \xi {\left(ru_{1}^{p}+\left(1-r\right)u_{2}^{p}\right)}^{1/p}+\xi {\left(\left(1-r\right)u_{1}^{p}+ru_{2}^{p}\right)}^{1/p}\leq h\left(r\right)\xi \left(u_{1}\right)+\left(1-h\left(r\right)\right)\xi \left(u_{2}\right)\\ & \; & +\left(1-h\left(r\right)\right)\xi \left(u_{1}\right)+h\left(r\right)\xi \left(u_{2}\right)-2l_{1}r\left(1-r\right){\left(u_{1}^{p}-u_{2}^{p}\right)}^{2}\\ & \; & =\xi \left(u_{1}\right)+\xi \left(u_{2}\right)-2l_{1}r\left(1-r\right){\left(u_{1}^{p}-u_{2}^{p}\right)}^{2}. \end{array}$$ (24)

*Multiply* (24) *by r*^*β*^ − 1 *and then integrate from* 0 *to* 1 *with respect to r, to get*

$$\begin{array}{c} \begin{array}{cc} & \int_{0}^{1}\left(r^{\beta -1}\times \xi {\left(ru_{1}^{p}+\left(1-r\right)u_{2}^{p}\right)}^{1/p}\right)dr+\int_{0}^{1}\left(r^{\beta -1}\times \xi {\left(\left(1-r\right)u_{1}^{p}+ru_{2}^{p}\right)}^{1/p}\right)dr\\ & \leq \xi \left(u_{1}\right)\int_{0}^{1}r^{\beta -1}dr+\xi \left(u_{2}\right)\int_{0}^{1}r^{\beta -1}dr-2l_{1}{\left(u_{1}^{p}-u_{2}^{p}\right)}^{2}\int_{0}^{1}\left(r^{\beta -1}\times r\times \left(1-r\right)\right)dr. \end{array} \end{array}$$ (25)

*Use* $s^{p}=\left(ru_{1}^{p}+\left(1-r\right)u_{2}^{p}\right)$ *in first integral of* (25), *and* $s^{p}=\left(\left(1-r\right)u_{1}^{p}+ru_{2}^{p}\right)$ *in second integral of* (25), *to get*

$$\begin{array}{c} \begin{array}{cc} & \int_{u_{1}}^{u_{2}}((\frac{u_{2}^{p}-s^{p}}{u_{2}^{p}-u_{1}^{p}}{)}^{\beta -1}\times \frac{ps^{1-p}\xi \left(s\right)}{\left(u_{2}^{p}-u_{1}^{p}\right)})ds+\int_{u_{1}}^{u_{2}}((\frac{s^{p}-u_{1}^{p}}{u_{2}^{p}-u_{1}^{p}}{)}^{\beta -1}\times \frac{ps^{1-p}\xi \left(s\right)}{\left(u_{2}^{p}-u_{1}^{p}\right)})ds\\ & \leq \frac{\xi (u_{1})}{\beta }+\frac{\xi (u_{2})}{\beta }-\frac{2l_{1}{\left(u_{1}^{p}-u_{2}^{p}\right)}^{2}}{\beta^{2}+3\beta +2}. \end{array} \end{array}$$

$$\begin{array}{c} \Rightarrow \frac{ps^{1-p}\Gamma \left(\beta \right)}{{\left(u_{2}^{p}-u_{1}^{p}\right)}^{\beta }}(M_{u_{1}^{+}}^{\beta }\xi \left(u_{2}\right)+M_{u_{2}^{-}}^{\beta }\xi \left(u_{1}\right))\leq \frac{\xi \left(u_{1}\right)+\xi \left(u_{2}\right)}{\beta }-\frac{2l_{1}{\left(u_{1}^{p}-u_{2}^{p}\right)}^{2}}{\beta^{2}+3\beta +2}. \end{array}$$ (26)

*From* (21) *and* (26), *we get*

$$\begin{array}{c} \begin{array}{cc} & \frac{1}{\beta }(\xi (\frac{u_{1}^{p}+u_{2}^{p}}{2}{)}^{1/p})\leq \frac{ps^{1-p}\Gamma \left(\beta \right)}{{\left(u_{2}^{p}-u_{1}^{p}\right)}^{\beta }}(\left(1-h\left(1/2\right)\right)M_{u_{1}^{+}}^{\beta }\xi \left(u_{2}\right)+h\left(1/2\right)M_{u_{2}^{-}}^{\beta }\xi \left(u_{1}\right))\\ & -\frac{l_{1}}{4}\;{\left(u_{1}^{p}-u_{2}^{p}\right)}^{2}\;\frac{\beta^{2}-\beta +2}{\beta^{3}+3\beta^{2}+2\beta },\\ & and\\ & \frac{ps^{1-p}\Gamma \left(\beta \right)}{{\left(u_{2}^{p}-u_{1}^{p}\right)}^{\beta }}(M_{u_{1}^{+}}^{\beta }\xi \left(u_{2}\right)+M_{u_{2}^{-}}^{\beta }\xi \left(u_{1}\right))\leq \frac{\xi \left(u_{1}\right)+\xi \left(u_{2}\right)}{\beta }-\frac{2l_{1}{\left(u_{1}^{p}-u_{2}^{p}\right)}^{2}}{\beta^{2}+3\beta +2}. \end{array} \end{array}$$

**Remark 4** *Choose h*(*r*) = *r and p* = 1 *in Theorem 3, to get the result for strongly convex function* (*see* [30]).

The H-H inequalities in context of Caputo Fractional derivatives for the strongly modified (*p*, *h*)-convex function is proved in following theorem.

**Theorem 4** *Let* ξ:[0,1]→R *be a strongly modified* (*p*, *h*)-*convex function with u*_1_ < *u*_2_ *for any u*_1_, *u*_2_ ∈ [0, 1], *then*

1n-βξn((u1p+u2p2)1/p)≤ps1-pΓ(n-β)(u2-u1)n-β((1-h(1/2))Mcu1+βξ(u2)+h(1/2)(-1)nMcu1−βξ(u1))-l1(n-β)4(u1p-u2p)2(4n-β+2+1n-β-4n-β+1),

*and*

ps1-pΓ(n-β)(u2p-u1p)n-β(Mcu1+βξ(u2)+(-1)nMcu1−βξ(u1))≤ξn(u1)+ξn(u2)-2l1(n-β)(u1p-u2p)2(n-β+1)(n-β+2).

**Proof 10** *Since ξ*^*n*^ *is strongly modified* (*p*, *h*)-*convex function therefore*,

$$\begin{array}{c} \xi^{n}({\left(\left(1-r\right)s^{p}+rw^{p}\right)}^{1/p})\leq \left(1-h\left(r\right)\right)\xi^{n}\left(s\right)+h\left(r\right)\xi^{n}\left(w\right))-l_{1}r\left(1-r\right){\left(s^{p}-w^{p}\right)}^{2}. \end{array}$$ (27)

*By taking r* = 1/2 *in* (27), *we get*

$$\begin{array}{c} \xi^{n}((\frac{s^{p}+w^{p}}{2}{)}^{1/p})\leq \left(1-h\left(1/2\right)\right)\xi^{n}\left(s\right)+h\left(1/2\right)\xi^{n}\left(w\right)-\frac{l_{1}}{4}{\left(s^{p}-w^{p}\right)}^{2}. \end{array}$$ (28)

*Assume* $s={\left(ru_{1}^{p}+\left(1-r\right)u_{2}^{p}\right)}^{1/p}$ *and* $l={\left(\left(1-r\right)u_{1}^{p}+ru_{2}^{p}\right)}^{1/p}$ *in* (28), *to get*

$$\begin{array}{ccc} & \; & \xi^{n}((\frac{u_{1}^{p}+u_{2}^{p}}{2}{)}^{1/p})\leq \left(1-h\left(1/2\right)\right)\xi^{n}({\left(ru_{1}^{p}+\left(1-r\right)u_{2}^{p}\right)}^{1/p})\\ & \; & h\left(1/2\right)\xi^{n}({\left(\left(1-r\right)u_{1}^{p}+ru_{2}^{p}\right)}^{1/p})-\frac{l_{1}}{4}{\left(u_{1}^{p}-u_{2}^{p}\right)}^{2}{\left(2r-1\right)}^{2}. \end{array}$$ (29)

*Multiply* (29) *by r*^*n*−*β*−1^ *and then integrate from* 0 *to* 1 *with respect to r, to get*,

$$\begin{array}{c} \begin{array}{cc} & \int_{0}^{1}({\left(r\right)}^{n-\beta -1}\times \xi^{n}(\frac{u_{1}^{p}+u_{2}^{p}}{2}{)}^{1/p})dr\\ & \leq \left(1-h\left(1/2\right)\right)\int_{0}^{1}\left({\left(r\right)}^{n-\beta -1}\times \xi^{n}{\left(ru_{1}^{p}+\left(1-r\right)u_{2}^{p}\right)}^{1/p}\right)dr\\ & +h\left(1/2\right)\int_{0}^{1}\left({\left(r\right)}^{n-\beta -1}\times \xi^{n}{\left(\left(1-r\right)u_{1}^{p}+ru_{2}^{p}\right)}^{1/p}\right)dr\\ & -\frac{l_{1}}{4}{\left(u_{1}^{p}-u_{2}^{p}\right)}^{2}\int_{0}^{1}\left({\left(r\right)}^{n-\beta -1}\times {\left(2r-1\right)}^{2}\right)dr. \end{array} \end{array}$$

$$\begin{array}{ccc} & \; & \Rightarrow \frac{1}{n-\beta }(\xi^{n}(\frac{u_{1}^{p}+u_{2}^{p}}{2}{)}^{1/p})\\ & \; & \leq \left(1-h\left(1/2\right)\right)\int_{0}^{1}\left({\left(r\right)}^{n-\beta -1}\times \xi^{n}{\left(ru_{1}^{p}+\left(1-r\right)u_{2}^{p}\right)}^{1/p}\right)dr\\ & \; & +h\left(1/2\right)\int_{0}^{1}\left({\left(r\right)}^{n-\beta -1}\times \xi^{n}{\left(\left(1-r\right)u_{1}^{p}+ru_{2}^{p}\right)}^{1/p}\right)dr\\ & \; & -\frac{l_{1}\times \left(n-\beta \right)}{4}\times {\left(u_{1}^{p}-u_{2}^{p}\right)}^{2}\times (\frac{4}{n-\beta +2}+\frac{1}{n-\beta }-\frac{4}{n-\beta +1}). \end{array}$$ (30)

*Assume* $s={\left(ru_{1}^{p}+\left(1-r\right)u_{2}^{p}\right)}^{1/p}$ *in first integral of* (30), *and* $s={\left(\left(1-r\right)u_{1}^{p}+ru_{2}^{p}\right)}^{1/p}$ *in second integral of* (30), *to get*

$$\begin{array}{ccc} & \; & \frac{1}{n-\beta }(\xi^{n}(\frac{u_{1}^{p}+u_{2}^{p}}{2}{)}^{1/p})\leq \left(1-h\left(1/2\right)\right)\int_{u_{1}}^{u_{2}}((\frac{u_{2}^{p}-s^{p}}{u_{2}^{p}-u_{1}^{p}}{)}^{n-\beta -1}\times \frac{ps^{1-p}\xi^{n}\left(s\right)}{u_{2}^{p}-u_{1}^{p}})ds\\ & \; & +h\left(1/2\right)\int_{u_{1}}^{u_{2}}((\frac{s^{p}-u_{1}^{p}}{u_{2}^{p}-u_{1}^{p}}{)}^{n-\beta -1}\times \frac{ps^{1-p}\xi^{n}\left(s\right)}{u_{2}^{p}-u_{1}^{p}})ds\\ & \; & -\frac{l_{1}\times \left(n-\beta \right)}{4}\times {\left(u_{1}^{p}-u_{2}^{p}\right)}^{2}\times (\frac{4}{n-\beta +2}+\frac{1}{n-\beta }-\frac{4}{n-\beta +1}). \end{array}$$ (31)

*Since*,

Mcu1+βξ(u2)=1Γ(n-β)∫u1u2(u2-s)n-β-1ξn(s)ds,Mcu1−βξ(u1)=(-1)nΓ(n-β)∫u1u2(s-u1)n-β-1ξn(s)ds.

*Therefore*, (31) *become*,

1n-βξ(u1p+u2p2)1/p≤ps1-pΓ(n-β)(u2-u1)n-β((1-h(1/2))Mcu1+βξ(u2)+h(1/2)(-1)nMcu1−βξn(u1))-l1(n-β)4(u1p-u2p)2(4n-β+2+1n-β-4n-β+1). (32)

*Also*, *ξ*^*n*^ *is strongly modified* (*p*, *h*)-*convex function, therefore*

$$\begin{array}{c} \xi^{n}({\left(ru_{1}^{p}+\left(1-r\right)u_{2}^{p}\right)}^{1/p})\leq h\left(r\right)\xi^{n}\left(u_{1}\right)+(1-h\left(r\right)\xi^{n}\left(u_{2}\right)-l_{1}r\left(1-r\right){\left(u_{1}^{p}-u_{2}^{p}\right)}^{2}, \end{array}$$ (33)

*and*

$$\begin{array}{c} \xi^{n}({\left(\left(1-r\right)u_{1}^{p}+ru_{2}^{p}\right)}^{1/p})\leq \left(1-h\left(r\right)\right)\xi^{n}\left(u_{1}\right)+h\left(r\right)\xi^{n}\left(u_{2}\right)-l_{1}r\left(1-r\right){\left(u_{1}^{p}-u_{2}^{p}\right)}^{2}. \end{array}$$ (34)

*By adding* (33) *and* (34), *we get*

$$\begin{array}{ccc} & \; & \xi^{n}({\left(ru_{1}^{p}+\left(1-r\right)u_{2}^{p}\right)}^{1/p})+\xi^{n}({\left(\left(1-r\right)u_{1}^{p}+ru_{2}^{p}\right)}^{1/p})\leq h\left(r\right)\xi^{n}\left(u_{1}\right)\\ & \; & +\left(1-h\left(r\right)\right)\xi^{n}\left(u_{2}\right)+\left(1-h\left(r\right)\right)\xi^{n}\left(u_{1}\right)+h\left(r\right)\xi^{n}\left(u_{2}\right)-2l_{1}r\left(1-r\right){\left(u_{1}^{p}-u_{2}^{p}\right)}^{2}\\ & \; & =\xi^{n}\left(u_{1}\right)+\xi^{n}\left(u_{2}\right)-2l_{1}r\left(1-r\right){\left(u_{1}^{p}-u_{2}^{p}\right)}^{2}. \end{array}$$ (35)

*Multiply* (35) *by r*^*n*−*β*−1^ *and then integrate from* 0 *to* 1 *with respect to r, to get*

$$\begin{array}{c} \begin{array}{cc} & \int_{0}^{1}\left(r^{n-\beta -1}\times \xi^{n}{\left(ru_{1}^{p}+\left(1-r\right)u_{2}^{p}\right)}^{1/p}\right)dr+\int_{0}^{1}\left(r^{n-\beta -1}\times \xi^{n}{\left(\left(1-r\right)u_{1}^{p}+ru_{2}^{p}\right)}^{1/p}\right)dr\\ & \leq \xi^{n}\left(u_{1}\right)\int_{0}^{1}r^{n-\beta -1}dr+\xi^{n}\left(u_{2}\right)\int_{0}^{1}r^{n-\beta -1}dr-2l_{1}{\left(u_{1}^{p}-u_{2}^{p}\right)}^{2}\int_{0}^{1}\left(r^{n-\beta -1}\times u_{1}\times \left(1-u_{1}\right)\right)dr. \end{array} \end{array}$$ (36)

*Use* $s={\left(ru_{1}^{p}+\left(1-r\right)u_{2}^{p}\right)}^{1/p}$ *in first integral of* (36), *and* $s={\left(\left(1-r\right)u_{1}^{p}+ru_{2}^{p}\right)}^{1/p}$ *in second integral of* (36), *to get*

$$\begin{array}{c} \begin{array}{cc} & \int_{u_{1}}^{u_{2}}((\frac{u_{2}^{p}-s^{p}}{u_{2}^{p}-u_{1}^{p}}{)}^{n-\beta -1}\frac{ps^{1-p}\xi^{n}\left(s\right)}{\left(u_{2}^{p}-u_{1}^{p}\right)})ds+\int_{u_{1}}^{u_{2}}((\frac{s^{p}-u_{1}^{p}}{u_{2}^{p}-u_{1}^{p}}{)}^{n-\beta -1}\frac{ps^{1-p}\xi^{n}\left(s\right)}{\left(u_{2}^{p}-u_{1}^{p}\right)})ds\\ & \leq \frac{\xi^{n}\left(u_{1}\right)}{n-\beta }+\frac{\xi^{n}\left(u_{2}\right)}{n-\beta }-\frac{2l_{1}{\left(u_{1}^{p}-u_{2}^{p}\right)}^{2}}{\left(n-\beta +1)(n-\beta +2\right)}. \end{array} \end{array}$$

⇒ps1-pΓ(n-β)(u2p-u1p)n-β(Mcu1+βξ(u2)+(-1)nMcu1−βξ(u1))≤ξn(u1)+ξn(u2)-2l1(n-β)(u1p-u2p)2(n-β+1)(n-β+2). (37)

*From* (32) *and* (37), *we get*

1n−βξnu1p+u2p21/p≤ps1−pΓ(n−β)(u2−u1)n−β(1−h(1/2))Mcu1+βξ(u2)+h(1/2)(−1)nMcu1−βξ(u1)−l1(n−β)4(u1p−u2p)24n−β+2+1n−β−4n−β+1,

*and*

ps1−pΓn−βu2p−u1pn−βMcu1+βξu2+−1nMcu1−βξu1≤ξnu1+ξnu2−2l1n−βu1p−u2p2n−β+1n−β+2.

## 6 Conclusion

The paper introduced the concept of strongly modified (*p*, *h*)-convex functions which generalizes the notion of strongly convex functions [19, 29] and provided a thorough examination of their properties. Furthermore, the study has explored Schur inequality and H-H inequalities for this new class of convexity. Some special cases of H-H inequalities are proved in [5, 18, 28–30]. Several illustrations and graphs have been demonstrated to check the validity of the proved inequalities. In future, it is possible to extend the H-H integral inequalities using fractional operators and fractional difference operators given in [31] for the obtained class of convexity.
